# Pressure and Temperature Spin Crossover Sensors with Optical Detection

**DOI:** 10.3390/s120404479

**Published:** 2012-04-10

**Authors:** Jorge Linares, Epiphane Codjovi, Yann Garcia

**Affiliations:** 1 GEMaC-CNRS, UMR 8635, Université de Versailles St Quentin en Yvelines, 45 Avenue des Etats Unis, Versailles Cedex 78035, France; E-Mails: jorge.linares@physique.uvsq.fr (J.L.); epiphane.codjovi@physique.uvsq.fr (E.C.); 2 Institute of Condensed Matter and Nanosciences, MOST–Inorganic Chemistry, Université Catholique de Louvain, Place L. Pasteur 1, Louvain-la-Neuve 1348, Belgium

**Keywords:** spin crossover, pressure sensors, optical detection, smart materials, sensitive paints

## Abstract

Iron(II) spin crossover molecular materials are made of coordination centres switchable between two states by temperature, pressure or a visible light irradiation. The relevant macroscopic parameter which monitors the magnetic state of a given solid is the high-spin (HS) fraction denoted n_HS_, *i.e.*, the relative population of HS molecules. Each spin crossover material is distinguished by a transition temperature *T*_1/2_ where 50% of active molecules have switched to the low-spin (LS) state. In strongly interacting systems, the thermal spin switching occurs abruptly at *T*_1/2_. Applying pressure induces a shift from HS to LS states, which is the direct consequence of the lower volume for the LS molecule. Each material has thus a well defined pressure value *P*_1/2_. In both cases the spin state change is easily detectable by optical means thanks to a thermo/piezochromic effect that is often encountered in these materials. In this contribution, we discuss potential use of spin crossover molecular materials as temperature and pressure sensors with optical detection. The ones presenting smooth transitions behaviour, which have not been seriously considered for any application, are spotlighted as potential sensors which should stimulate a large interest on this well investigated class of materials.

## 1. Introduction

There is a current need to develop solid state sensors for temperature and pressure that could find various applications in industrial sectors seeking quality testing of final products at the end of chain productions [1] or for direct or remote sensing applications. It concerns not only the aerospace [2], aeronautics, plastics and (ship)building industry, to name a few flourishing areas, but also the nuclear power domain [3] and the automotive sector, which is considered as one of the most important economic ones by revenue. Although pressure sensing is less used than temperature sensing (for thermometers and time temperature integrators [4,5]), this physical parameter has become more and more recognized as an important control factor in various fields, e.g., in soil sciences [6,7] and for marine applications [8]. Various sensitive elements and transducers already meet range, sensitivity, linearity and other requirements for a specific use [9]. Some can sense temperature and pressure thanks to two sensors arranged on a device [10], but when very small size sensors or when a large number of sensors are required, novel concepts must be conceived. Detecting two thermodynamic parameters, such as temperature and pressure, using the very same sensor in a single device would be advantageous for novel applications, saving measurement time and space, and offering prospects in the area of miniaturization. Such sensors do not exist yet on the market, but the attractive class of iron(II) spin crossover (SCO) molecular materials could be unique candidates for this purpose since these inorganic compounds can be switched between two stable states by both temperature and pressure [11–14]. These multifunctional materials can in addition be miniaturized as nanoparticles [15,16], nanostructured by unconventional wet [17] and soft lithography methods [18–20], and deposited on devices as thin films [21]. We propose herein the use of SCO molecular materials as pressure and temperature sensors with optical reflectivity detection. After a general description of the SCO phenomenon, we will discuss methods and requirements for different families of materials, and end up by suggesting some specific applications where these sensor materials could be used.

## 2. Spin Crossover Phenomenon

### 2.1. Spin Crossover Occurrence

**Scheme 1.**
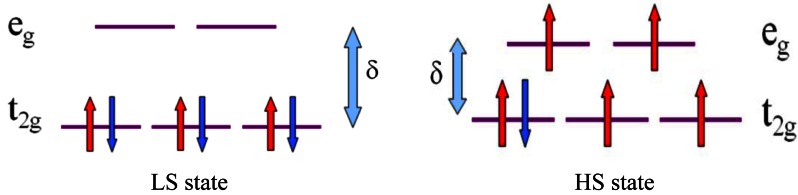
Electronic distribution for a 3d^6^ ion in *O_h_* symmetry being in the LS and HS states. δ stands for the ligand field splitting.

At the electronic level, the ligand field strength δ acting on the iron centre of an iron(II) complex raises the degeneration of the 3d orbitals, thus affording in an octahedral field, two orbitals (e_g_) separated from the other three orbitals (t_2g_) (Scheme 1).

According to relative values of δ and the electrons pairing energy ∏, the ground state can be defined as follows:

- When δ ≫ ∏, all six electrons occupy the three orbitals of the lowest energy. The total spin is S = 0 and the iron(II) ion is in the low-spin (LS) state.
- If δ ≪ ∏, five electrons occupy all the five orbitals and the sixth electrons will be in one of the lowest energy orbital. The total spin S = 2 and the iron(II) ion is in the high-spin (HS) state.

**Figure 1.**
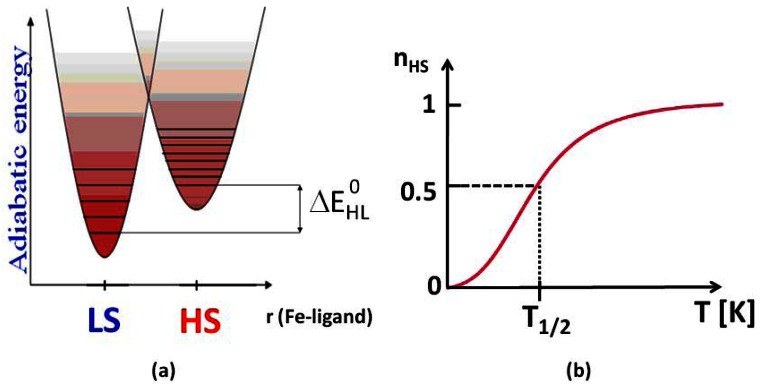
(**a**) Jablonski diagram for SCO molecules with adiabatic energy *vs*. Fe-ligand distance; (**b**) n_HS_ *vs*. T profile for a gradual SCO behaviour.

Figure 1(a) shows the molecular configuration diagram, *i.e.*, a plot of adiabatic energy *vs.* the distortion coordinate, here the metal-ligand distance, which is believed to be the most perturbed coordinate upon the spin transition.

Thermally induced SCO conditions are met in the intermediate case when the energy difference between the first vibronic levels of the LS and HS states, ΔE_HL_^0^, ranges in the order of thermal energy, k_B_T [11]. The Fe-ligand bond length increases to about 10%, on going from the LS to the HS state [11]. Other physical properties that are modified by the SCO phenomenon are:

- The magnetic properties from a diamagnetic state (*S* = 0) to a paramagnetic state (*S* = 2).
- The colour leading to thermochromism, photochromism or piezochromism. For instance, most iron(II) 1,2,4-triazole/tetrazole complexes are red purple in the LS state and colourless in the HS state making them usable in display devices [22–24].
- The frequencies of vibrations, and thus the entropy which increases during the LS to HS switch.

### 2.2. Thermodynamics Considerations

The principle of the thermal SCO behaviour can be understood on thermodynamic grounds. The molecular free energy *ΔG* variation during the LS-HS transformation for non-interacting molecules is given by:

(1) ΔG=ΔH−TΔS

(2) $$\text{where}\;\Delta \text{G}={\text{G}}_{\text{HS}}-{\text{G}}_{\text{LS}}$$

(3) $$\text{and}\;\Delta \text{H}={\text{H}}_{\text{HS}}-{\text{H}}_{\text{LS}}$$

(4) $$\text{and}\;\Delta S={\text{S}}_{\text{HS}}-{\text{S}}_{\text{LS}}$$

The temperature for which the number of SCO active molecules is in a 50% ratio, is called the transition temperature *T_1/2_* and is defined by ΔG = 0, which implies:

(5) $$T_{1/2}=\Delta \text{H}/\Delta \text{S}$$

*ΔS* is related to the degeneracies ratio g = g_HS_/g_LS_ by the relation:

(6) $$\Delta S=\text{R}\ln({\text{g}}_{\text{HS}}/{\text{g}}_{\text{LS}})=\text{R lng}$$

A simple description of a SCO between LS and HS states can be given: below *T*_1/2_, the enthalpy factor dominates, *ΔG* > 0 and the LS state is the most stable. As far as temperature increases, the entropic factor *TΔS* increases and above *T_1/2_* the entropy factor dominates, *ΔG* < 0 and the HS state becomes more stable. The entropy term is so strong that the population of the LS state is almost zero.

The system is in general described by the HS fraction, usually denoted n_HS_ or γ_HS_ which represents the proportion of molecules in the HS state.

We can also estimate the pressure effect on *T*_1/2_: under pressure the free energy *ΔG* is written as:

(7) ΔG=ΔH−TΔH+PΔV

It results that the pressure dependence of *T_1/2_* is:

(8) $${\text{T}}_{1/2}(\text{P})={\text{T}}_{1/2}(0)+\text{P}\Delta \text{V}/\Delta \text{S}$$

For interacting molecules an interaction parameter *Γ* is introduced:

(9) $$\Delta G=\Delta H-T\Delta S+n_{HS}(1-n_{HS})\Gamma$$

According to the strength of the *Γ* parameter, different thermal behaviours for n_HS_(T) are obtained. At low temperature, the LS state is the more stable one, which means n_HS_ = 0. When the temperature is increased, the HS state is populated. Figure 1(b) is obtained for non-interacting molecules resulting in a gradual spin conversion/spin equilibrium as observed in the liquid state or for diluted materials, e.g., [Fe_1-x_Zn_x_(2-picolylamine)_3_]Cl_2_·EtOH [13]. In the solid state and in the presence of interactions of elastic and electronic origin between the spin changing molecules, a steep phase transition can result, which is termed spin transition (ST). For strongly interacting systems, the ST can be accompanied by a thermal hysteresis loop which is the sign of a first order transition that can be observed provided interactions exceed a threshold value Γ_c_, as observed for instance for the 1D chain [Fe(4-amino-1,2,4-triazole)_3_] (NO_3_)_2_ [25]. In this case, critical temperatures T_c_^↑^ and T_c_^↓^ can be used to define the transition temperatures. A quite unusual case is when the transition occurs stepwise which may results for instance from the presence of two crystallographic lattice sites, as observed for [Fe(4,4′-bis-1,2,4-triazole)_3_] (ClO_4_)_2_ [26] or intermediate magnetic phases [11]. A spin conversion can also occur with a residual effect at low temperatures which is typical when crystal defects are present, for instance as a result of grinding [11] or when chains of different lengths are identified, for instance for coordination polymers [27]. This phenomenon can also be observed at higher temperatures. Basically, a combination of these curves can also be theoretically predicted. Most interestingly, similar shape curves can be observed when the trigger is pressure [28].

### 2.3. Ising-Like Model for Spin Crossover Molecules

On the basis of the pioneering theoretical work of Wajnsflasz and Pick [29], a Ising-like model for considering the n_HS_ evolution has been proposed [30–32]. In this phenomenological two-level model, a fictitious spin operator *σ* is introduced with two eigen values: *σ* = ± 1 for HS and LS states, respectively with respective degeneracies *g_HS_* and *g_LS_*.

For non-interacting molecules the Hamiltonian is:

(10) $$\hat{H}=\sum_{i=1,N}\frac{\Delta }{2}{\hat{\sigma }}_{i}$$

where *Δ* is the electronic gap between LS and HS states, which was labelled as δ in the previous section. The thermal average value of *σ* is:

(11) $$\langle \sigma \rangle =\tanh\left(-\frac{\Delta -k_{B}T\ln(g)}{2k_{B}T}\right)$$

The HS fraction n_HS_ is related to <σ> as follow:

(12) $$n_{HS}=\frac{1+\langle \sigma \rangle }{2}$$

The model is termed “like” because the ratio *g* may be quite large, up to a few thousands, as it involves both the spin degeneracies and the density of vibrational states [33]. This model can also be viewed as a simple Ising model under a temperature-dependent “effective” field which accounts for the different degeneracies of the levels.

Among the various SCO molecules available, 1D coordination polymers play a growing role as this family of molecules not only can display highly cooperative spin transitions but also can be combined to several other properties in hybrid materials (e.g., SCO and liquid crystal properties [34]). In these molecules short and long range interactions are recognized as being critical to leads to highly cooperative materials [27]. The 1D Hamiltonian for interacting molecules including long- and short-range interactions is given by ([31]):

(13) $$\hat{H}=\sum \frac{\Delta_{\text{eff}}-G\langle \sigma \rangle }{2k_{B}T}-\text{J}\sum \sigma_{\text{i}}\sigma_{i+1}$$

where *J* and *G* are the short and long range interactions, respectively, and *Δ_eff_* = *Δ* − *k_B_ T* ln *g*.

**Figure 2.**
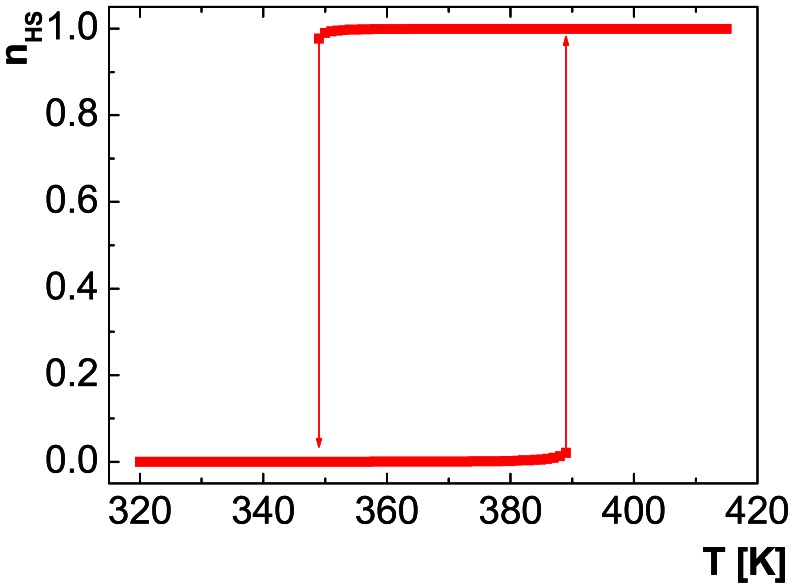
Simulated HS fraction *vs.* temperature for [Fe(Htrz)_2_trz]BF_4_.

With *Δ* = 3127 K, *J* = 800 K, *G* = 105.5 K, ln g = 8.448 and using the Hamiltonian given by equation (13), we obtain the temperature dependence of *n_HS_* shown in Figure 2. This result fits the experimental data obtained for the 1D chain compound [Fe(Htrz)_2_trz]BF_4_ (Htrz = 4-H-1,2,4-triazole, trz = 1,2,4-triazolato) [35], a material which is currently the topic of intense miniaturization efforts and considered for implementation in logistic devices [36–41].

Taking into account the pressure *P* that can be applied on the material, *Δ_eff_* now can be expressed by:

(13) $$\Delta_{\text{eff}}=\Delta +\alpha P-k_{B}T\ln g$$

**Figure 3.**
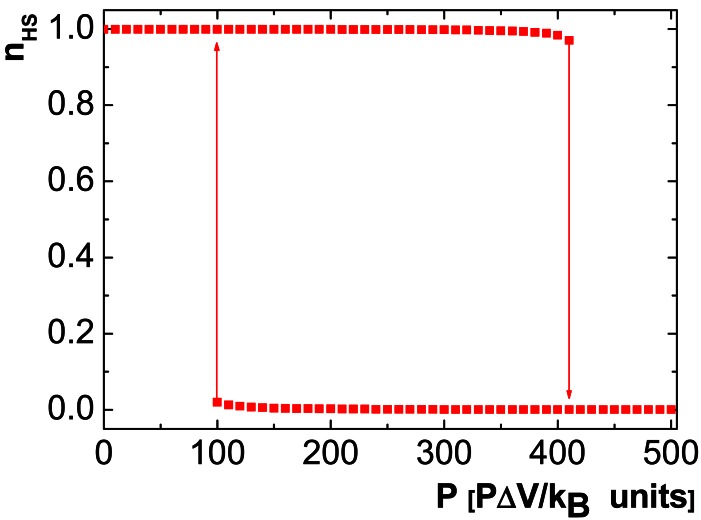
Simulated behaviour of n_HS_ *vs.* P at 400 K for [Fe(Htrz)_2_trz]BF_4_. ΔV corresponds to the volume difference between HS and LS states which has been recently evaluated as 129.1 Å^3^ by high resolution synchrotron X-ray powder diffraction [42].

This way, the HS fraction now *vs.* pressure at *T* = 400 K for the 1D chain compound [Fe(Htrz)trz]BF_4_ can be simulated, revealing a square shape hysteresis loop (Figure 3).

Note that this curve has not yet been experimentally observed on [Fe(Htrz)_2_trz]BF_4_, but is here predicted in this work to demonstrate the usefulness of the Ising-like model in the design of future pressure sensors, and encourage its use to future users.

## 3. Spin Crossover Sensors

### 3.1. General Considerations

**Scheme 2.**
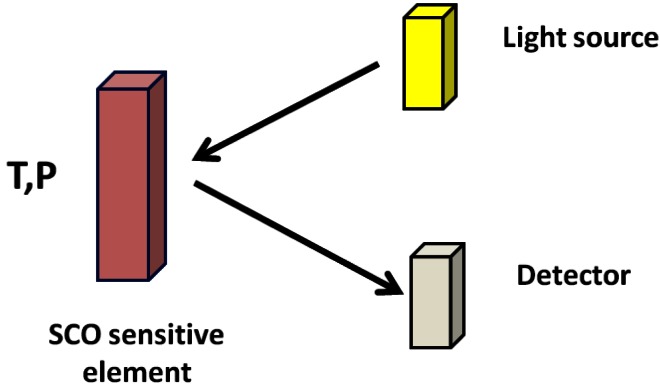
Principle of a SCO sensor based device with optical reflectivity detection.

Each SCO material is characterized by well defined *T*_1/2_ and *P*_1/2_. Applying pressure induces a shift from HS to LS state, which is, the direct consequence of the lower volume for the LS molecule [43], although the reverse situation could also be observed [44]. In both cases, the change from LS to HS is easily detectable by optical means because SCO molecular materials often display thermochromism that is a different colour in the HS and LS states. This is the case for instance for the family of 1D ST chain compounds with 1,2,4-triazole or 1-tetrazole ligands that are deep purple powders in the LS state and white in the HS state [22], and that present the best contrast available today. Here we propose the use of SCO molecular materials with optical reflectivity detection as an alternative for a new range of temperature and pressure sensors [45-47]. The sensor material is deposited either as a thin film or nanostructured by soft-lithography on a sensitive element. A light source and a detector are used within an optical reflectivity set-up (Scheme 2).

One of the attractive aspects of these materials is their pronounced colour change that makes possible the visual detection of the variation of a measured parameter that can alert a given user without the use of sophisticated electronics such as optical detectors or radio frequency identification tags to record experimental data, which can be useful in remote control applications. On the other hand, due to a well defined (*T*, *P*) phase diagram [48], the same element can be used to measure temperature at constant pressure and pressure at a constant temperature, which is particularly appealing. But one should take care of the measurement error due the fluctuation of one parameter when detecting the other. An estimation of such error can be made according to the c.a 20 K/kbar rate variation of *T*_1/2_ *vs. P*_1/2_ [48]. Thus, when using SCO materials as a temperature sensor, 1 bar error on pressure will only induce 20 mK on the measured temperature. On the other hand, when using the material as a pressure sensor, 1 K error on temperature will induce 50 bars error on the measured pressure; this error leads to a low relative error in high pressure range. The sensitivity and resolution of SCO sensors also depend on the contrast of the colour change of the materials which can be high when all molecules of the materials are concerned by the switching process [48]. Spatial resolution, repeatability and time response are the biggest advantages of SCO sensors due to the molecular origin of the phenomenon and its associated non fatigability [23]. Most interestingly, the pressure range necessary to switch a SCO material can be tailored through chemical design.

### 3.2. Examples of Sensors Using SCO Elements

Many iron(II) compounds exhibit sharp transitions with dramatic modification of the magnetic moment with temperature, sometimes with hysteresis necessary for memory use [22]. Those with smooth spin conversion behaviour have a quasi linear response and seem however to be the most simple materials to implement. We describe below a few possible methods depending on the SCO curve of the selected materials.

#### 3.2.1. Method for Materials Presenting a Sharp Spin Transition without Hysteresis

**Scheme 3.**
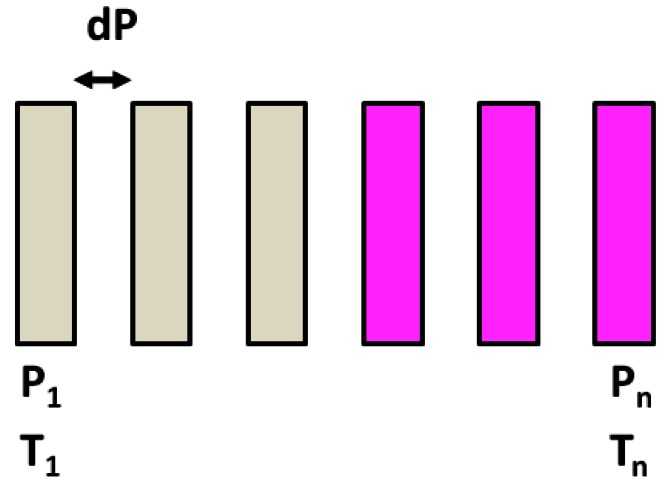
View of a pressure (or temperature) sensor device using n elements filled by a given ST compound. Colours are given for a pressure sensor device.

Iron(II) compounds presenting a sharp ST without hysteresis can be obtained by chemical design through mastering elastic interactions in the crystalline state [26]. The proposed device contains n sensor elements with *T*_1/2_ or *P*_1/2_ ranging from *T_1_* (*P_1_*) to *T_n_* (*P_n_*) with optimal resolution d*P*, in the range P_n_-P_1_ (Scheme 3). No measurement is possible out of the range. When a given pressure *P* is applied to the device, all sensor elements having *P*_1/2_ ≤ *P* change their colour and those having *P*_1/2_ > P keep their original colour. The measured pressure *P* is the *P*_1/2_ of the upmost element having changed its colour. This colour change disappears when pressure is released and allows new measurements. For a few known materials, this change can be irreversible, which makes them suitable for threshold detection [49], which can be useful for instance for ergonomic applications. An interface pressure sensor using such materials could be proposed in order to prevent pressure ulcers in the seats in hospital or in cars and planes. For such applications, ST compounds requesting low pressure would be best suited.

#### 3.2.2. Method for Compounds Presenting a Smooth Spin Conversion without Hysteresis

A very large number of materials display a smooth spin conversion curve without hysteresis, as shown in Figure 1(b). This behaviour is characteristic of most mononuclear complexes that represent the widest family of SCO materials investigated up to now [11,14,50–53]. In most cases, these materials are not studied in many details, mainly in term of pressure effects, if not discarded right after synthesis and even not published due to their gradual SCO nature. However, these materials could be useful for a pressure sensing application, thus opening novel application perspectives in the SCO field. The output voltage V from the optical detector of one single element sensor varies linearly with the temperature *T* or pressure *P*. Only calibration at both ends of the range with *T* or *P* standards is needed to deduce *T* and *P*, as no hysteresis is detected. Sensitivity should be tuned by undertaking syntheses to optimize both linearity and colour contrast.

#### 3.2.3. Method for Compounds Presenting an Abrupt Transition with Hysteresis of Width *ΔP*

Some materials present an abrupt ST accompanied by a hysteresis of width *ΔP* [54]. This family of materials can be useful to detect differential pressures. When a system set at a given pressure *P* in the middle of the pressure hysteresis loop undergoes a variation *ΔP*/2, the colour of the compound changes. The sensitivity of such pulse detector depends on *ΔP*. The family of 2D coordination polymers [Fe(3-Fpy)_2_M(CN)_4_] M^II^ = Ni, Pd, Pt, with ΔP ∼ 0.1 GPa, which operates at room temperature with a distinct colour change from pale yellow (HS) to red (LS), fulfill these criteria [55]. This is also the case for the model ST compound [Fe(phen)_2_(NCS)_2_] (polymorph II) whose magnetic properties were investigated under pressure at variable temperature revealing a spin state change from HS to LS at room temperature [56], which was later studied by optical reflectivity [57]. The 1D coordination polymer [Fe(hyptrz)_3_] (4-chlorobenzenesulfonate)_2_·H_2_O whose hysteretic and thermochromic ST is shifted above room temperature by hydrostatic pressure could also be considered in this section [58]. Similary, the abrupt ST occurring for the chromium(II) complex, [CrI_2_(1,2-bis(diethylphosphino)ethane)_2_] which can be switched at room temperature and changes colour from purple brown (HS) to brilliant violet (LS) could be studied too [56,59].

## 4. Conclusions

Prototypes using SCO materials as temperature and pressure sensors have been presented with possible implementation of these elements in pressure devices. Several possible applications based on SCO sensors could be considered and are only limited by our imagination. We could think for instance of functional paints containing pressure sensitive particles that could cover a whole surface of a vehicle or military units used either to write a given information (e.g., a plane or a submarine varying its colour at a given altitude or depth, respectively) or indicate the pressure. This latter option could be particularly useful for the underwater diving equipment industry (e.g., marine and speleology applications). These applications need low pressure sensors and presumably new materials to be synthesised. In this respect, it is interesting to note that inks containing ST materials have been proposed to protect selected items against forgery (banknotes, compact disks, credit cards, tax seals for tobacco goods, …). These functional papers use small pressure to address the information and visual detection to read it [60]. We have restricted ourselves to two physical parameters but applications could be extended to the use of light absorption [11,61] or fluorescence of these materials [62–64], as well as their gas sensing properties [65–67]. From a SCO point of view, this contribution also illustrates the usefulness of materials exhibiting smooth spin conversion curves which up to now have not been considered for potential applications. This attractive perspective could stimulate further syntheses, investigations and a renew interest of this well investigated class of substance.
